# Chloro-nitrous Oxide

**Published:** 1869-05

**Authors:** H. L. Sage


### CHLORO-NITROUS OXIDE.

BY H. L. SAGE.

I saw an article in the February number of the REGISTER, under the above heading, being a paper read at the Michigan Dental Association, in which the writer calls attention to the discovery of "a new anæsthetic agent," prepared by "mixing with nitrous oxyd a certain proportion of the vapor of chloroform." He speaks of "six months exclusive use" of this agent by the discoverer, H. W. P. Barker. Permit me to call attention to the fact that I made use of this combination at least one year ago. In this case I mixed with six gallons of nitrous oxyd about three drops of chloroform, and administered a portion of it to a strong, robust man. It induced anæsthesia in one quarter of the usual time—almost immediately—the effect being so rapid that it startled me. The results were not pleasant, nausea and prostration being produced in such a degree that I abandoned its use.

